# Wild *Lathyrus*—A Treasure of Novel Diversity

**DOI:** 10.3390/plants13213028

**Published:** 2024-10-29

**Authors:** Akanksha Singh, Rind Balech, Surendra Barpete, Priyanka Gupta, Outmane Bouhlal, Sawsan Tawkaz, Smita Kaul, Kuldeep Tripathi, Ahmed Amri, Fouad Maalouf, Sanjeev Gupta, Shiv Kumar

**Affiliations:** 1 International Center for Agricultural Research in the Dry Areas (ICARDA), New Delhi 110012, India; 2 International Center for Agricultural Research in the Dry Areas (ICARDA), Terbol 1108-2010, Lebanon; 3 International Center for Agricultural Research in the Dry Areas (ICARDA), Amlaha 466113, India; 4 Département de Phytologie, Institut de Biologie Intégrative et des Systèmes Pavillons Charles-Eugène Marchant, Université Laval, Québec, QC G1V 4G2, Canada; 5 International Center for Agricultural Research in the Dry Areas (ICARDA), Rabat 10112, Morocco; 6 International Center for Agricultural Research in the Dry Areas (ICARDA), 2 Port Said, Victoria Square, Maadi, Cairo 11140, Egypt; 7 Division of Germplasm Evaluation, ICAR-National Bureau of Plant Genetic Resources, New Delhi 110012, India; 8 Crop Science Division, Indian Council of Agricultural Research, Krishi Bhawan, New Delhi 110001, India

**Keywords:** *Lathyrus*, grasspea, CWRs, pre-breeding, germplasm conservation and utilization

## Abstract

Grasspea (*Lathyrus sativus* L.) is a climate-smart legume crop with adaptation to fragile agroecosystems. The genus *Lathyrus* is recognized for its vast genetic diversity, encompassing over 160 species, many of which are cultivated for various purposes across different regions of the world. Among these, *Lathyrus sativus* is widely cultivated as food, feed, and fodder in South Asia, Sub-Saharan Africa, and the Central and West Asia and North Africa (CWANA) regions. Its global cultivation has declined substantially due to the stigma posed by the presence of neurotoxin β-N-oxalyl-L-α, β-diaminopropionic acid (β-ODAP) in its seeds and green foliage. Overconsumption for a longer period of grasspea seeds harvested from landraces may lead to a neurological disorder called neurolathyrism in humans. ODAP is an obstacle for grasspea expansion, but crop wild relatives (CWRs) have been found to offer a solution. The incorporation of CWRs, particularly *Lathyrus cicera*, and landraces into breeding programs may reduce the ODAP content in grasspea varieties to a safer level. Recent advances in genomics-assisted breeding have expanded the potential for utilizing challenging CWRs to develop grasspea varieties that combine ultra-low ODAP levels with improved yield, stability, and adaptability. Further progress in omics technologies—such as transcriptomics, proteomics, and metabolomics—along with genome sequencing and editing, has greatly accelerated the development of grasspea varieties with reduced or zero ODAP content, while also enhancing the plant’s agronomic value. This review highlights the significance of utilizing CWRs in pre-breeding programs, and harnessing advanced tools and technologies to enhance the performance, adaptability, and resilience of grasspea in response to changing environmental conditions.

## 1. Introduction

Grasspea *(Lathyrus sativus* L.) is a diploid legume (2n = 14), domesticated in the Balkan peninsula [1]. It belongs to the family *Leguminosae*, the subfamily *Papilionoideae*, and the tribe *Vicieae*. *Grasspea* is primarily a self-pollinated crop, but outcrossing, to the extent of 9–25%, is reported depending on flower color and size, and pollinators [2]. It thrives on poor soils in semi-arid and arid environments and exhibits remarkable tolerance to biotic and abiotic stresses [3]. It is highly efficient at fixing atmospheric nitrogen through symbiotic relationships with Rhizo-bacteria and contributes ~108–125 kg/ha of nitrogen to the soil [4,5]. Cultivating grasspea in rotation with cereals reduces the nitrogen requirements of subsequent crops, making it a crucial element of sustainable farming systems [6]. Grasspea is cultivated in many countries across Asia (India, Pakistan, Bangladesh, Nepal, and China), Southern Europe (France, Portugal, Italy, and Spain), and Africa (Ethiopia, Eritrea, Ghana, Sudan, Niger, Ivory Coast, and Mauritania). It serves as a protein source for both humans and animals. In certain regions, including North America, South America, Australia, and Europe, grasspea finds use in fodder production [7]. Additionally, in both Europe and North America, it is grown as a cover crop and a green manure crop to prevent soil erosion [8,9,10]. Grasspea holds good potential as a legume crop for low-rainfall parts of the Canadian prairies and low-quality soils in South Australia and Central Asia [8,9,10].

Nutrient-rich food crops with reduced water demands such as grasspea hold an important place in the food system to mitigate global malnutrition. Grasspea ranks as the second-richest protein source after soybean and it also contains L-homoarginine, which improves cardiac function [11]. While the crop possesses numerous favorable agronomic and nutritional qualities, the primary barrier preventing it from becoming the first choice among legumes is the presence of an anti-nutritional factor, b-N-oxalyl-L-α, β-diaminopropionic acid (β-ODAP), in most of the plant’s tissues, including its seeds. The prolonged consumption of β-ODAP causes neurolathyrism, a neurological condition in humans that results in irreversible paralysis of the lower limbs [12,13]. The presence of β-ODAP has led to reduced acreage under grasspea cultivation and even to a ban on the sale and cultivation of grasspea in some countries, and to it being given the status of an “orphan crop” [14]. Despite such bans, the potential of grasspea for research and future agricultural use cannot be hidden. The primary area of research on grasspea is developing varieties with low or zero β-ODAP content. To reach this goal, plant breeders have developed cultivars with low β-ODAP, such as Mahateora, Ceora, LS8246, BioL-212, Bari Khesari-1, and Prateek [15]. Despite the efforts, no cultivar has been developed with zero β-ODAP content to date. Efforts have been made to understand the biosynthesis and genetics of β-ODAP, but there are still significant gaps in our knowledge of the production β-ODAP in grasspea. Widespread use of grasspea cultivation is also constrained by a few other undesirable agronomic characteristics, such as pod shattering, its prostrate-spreading plant habit, late maturity, and indeterminate development, that require improvement [16].

The development of high-yielding grasspea varieties with zero/low seed ODAP content and an early maturity is needed to make the crop significantly more suitable for human consumption and expand its cultivation, particularly in dry regions. To facilitate the continued development of new cultivars, CWRs serve as a valuable source of diversity. Breeders often face one of the most difficult challenges when searching for useful genetic diversity in elite germplasm, particularly economically important traits.

However, wild *Lathyrus* holds an extensive reservoir of valuable traits, including tolerance to abiotic stresses, resistance to diseases, and low ODAP content, which can significantly enhance the resilience, yield, and nutritional quality of cultivated grasspea. Despite this great potential, wild *Lathyrus* remains largely underutilized in breeding programs due to challenges such as reproductive barriers, lack of effective pre-breeding efforts, and underdeveloped genomic resources. The emergence of high-throughput genotyping and phenotyping technologies, along with advancing biotechnologies, offers new opportunities to explore genetic variation. These can be leveraged for the development of enhanced grasspea lines with zero/low ODAP content. This review aims to fill this gap by providing a comprehensive synthesis of the genetic diversity present in wild species, identifying key reproductive and technological challenges, and outlining modern tools like genome sequencing, omics approaches, and genome editing that can unlock this diversity for breeding. By addressing these gaps, the review will offer a roadmap for utilizing wild *Lathyrus* to drive crop improvement and ensure greater food security in challenging environments.

## 2. Origin, Domestication, and Geographic Distribution

The genus *Lathyrus* exhibits a wide range of diversity, with its species distributed across Europe, Asia, North America, South America, and East Africa. However, the primary center of diversity is concentrated in the Mediterranean and Irano-Turanian regions [17]. While the genus is naturally adapted to temperate regions, it can also be found at high altitudes in tropical Africa. It is worth noting that the genus also includes numerous restricted endemic species, which are distributed across all continents except Australia and Antarctica [17]. The eco-geographic distribution of all but a few *Lathyrus* species is poorly understood, particularly for those in the section *Notolathyrus* that are endemic to South America. There is a need for a detailed eco-geographic study of the whole genus if it is to be effectively and efficiently conserved and utilized for grasspea improvement. The most widely cultivated species for human consumption is *L. sativus*. Other species grown for forage and/or grain include *L. cicera*, *L. ochrus*, *L. clymenum*, *L. tingitanus*, *L. latifolius*, and *L. sylvestris* [18]. *Lathyrus cicera* is cultivated in Greece, Cyprus, Iran, Iraq, Jordan, Spain, and Syria and *L. ochrus* in Cyprus, Greece, Syria, and Turkey [19]. *Lathyrus hirsutus* and *L. clymenum* are cultivated as minor forage or fodder in the southern United States and Greece [20]. A few species such as *L. odoratus*, *L. latifolius*, and *L. sylvestris* are grown as ornamental crops [21].

Grasspea originated in southwest and central Asia and later expanded into the eastern Mediterranean region [1]. However, phytogeographical and archaeobotanical data indicate that its origin on the Balkan peninsula dates back to the 6th millennium BC [1]. Vavilov [22] described Central Asia and Abyssinia as the centers of origin for grasspea. The important archaeological evidence of grasspea is noted in adjacent areas of Tepe Sadz (7500–5700 BCE) and Ali Kosh (9500–7600 BC) in Iran [23] and in the Gangetic plains of India (2000–1500 BCE) [24] with presumption of its introduction from West Asia. The oldest fossils of grasspea utilized for human consumption were discovered in the Euphrates valley during the middle pre-pottery Neolithic era (8200–7500 BCE) [25]. Evidence of grasspea cultivation has been discovered at Bronze Age sites in India, Greece [26], and Ethiopia [27]. In the Fertile Crescent, Eastern Europe, and the Mediterranean region, a multitude of Neolithic archaeological sites have discovered fossilized seeds dating back to 6000 BCE of *L. sativus* L. and related species like *L. cicera* L. and *L. ochrus* L. DC. [1,28,29]. The true natural distribution of *L. sativus* is heavily concealed by the effects of cultivation, which complicates the exact identification of its center of origin. The absence of significant morphological distinctions between wild and domesticated populations likely stems from the fact that *L. sativus* has historically served a dual purpose, both as a source of grain and forage, in its native regions. Within this context, small-seeded accessions represent the more primitive types with hard seeds. In contrast, the selection for forage use has given rise to local varieties characterized by larger leaves, pods, and seeds, although these tend to have lower seed yields, particularly in the Mediterranean region.

## 3. Taxonomic Classification and the Gene Pool

*Lathyrus* belongs to the tribe *Vicieae* of the subfamily *Papilionoideae*, along with other genera that are *Vicia*, *Lens*, *Pisum*, and *Vavilovia*. The exact differences between *Lathyrus* and *Vicia* are poorly defined; however, oroboid species serve as a bridge between both genera [17]. Schaefer et al. [30] suggested that the Fabeae tribe originated in the Eastern Mediterranean during the middle Miocene. From this region, the tribe expanded its range across Eurasia, into Tropical Africa, and made at least seven transoceanic migrations to the Americas via the Atlantic and Pacific. The most likely explanation for these Atlantic crossings appears to be long-distance dispersal events, as Schaefer et al. [30] ruled out the possibility of ancient steppingstone dispersal through the Atlantic islands. The authors, based on phylogenetic data, indicated that the genus *Lathyrus* is not monophyletic and suggested that a more accurate classification of the Fabeae tribe should incorporate *Pisum* and *Vavilovia* as well. This reclassification is further supported by the whole-plastid genomes currently available for *L. sativus* and *P. sativum* [31]. Additionally, the genera *Pisum* and *Lathyrus* both produce the phytoalexin pisatin, a compound absent in *Vicia* and *Lens* [32]. Recent taxonomic revisions have proposed placing *Pisum sativum* within the *Lathyrus* genus [33], though this reclassification has been questioned [34]. These conflicting perspectives highlight the need for further comprehensive studies, including additional molecular data and phylogenetic analyses, to clarify the relationship between these genera and ensure a more accurate classification.

The genus *Lathyrus* contains approximately 187 species [35,36]. Kupicha [37] divided the genus into 13 sections based on the taxonomic classification, but more research is needed to establish the phylogenetic relationships among the sections and species (Table 1). *Lathyrus* species have been divided further into five groups, namely, *Nissolia*, *Aphaca*, *Cicerula*, *Clymenum*, and *Lathyrus.* The *Lathyrus* group includes perennial species, whereas the other four groups are annual [37,38,39]. Asmussen and Liston [38] provided a comprehensive summary of the taxonomic identification and evolution of the genus *Lathyrus* based on morphological characterization. *Lathyrus* has been further classified into primary, secondary, and tertiary gene pools based on crossability [23,40,41,42]. The primary gene pool includes grasspea landraces and cultivars, and the secondary gene pool includes different species such as *L. cicera*, *L. pseudocicera* Pamp., *L. amphicarpus* L., *L. gorgoni* Parl., *L. marmoratus* Boiss. & Balansa, *L. blepharicarpus* Boiss., *L. chloranthus* Boiss. & Balansa, *L. chrysanthus* Boiss., *L. hierosolymitanus* Boiss., and *L. hirsutus* L. There is limited information available regarding the cross-compatibility of these species with cultivated *L. sativus*. However, Heywood et al. [43] noted that certain species, including *L*. *chrysanthus*, *L*. *gorgoni*, *L. marmoratus*, and *L. pseudocicera*, which belong to the secondary gene pool, are compatible with *L. sativus*, though they only produce ovules. The remaining species are kept in the tertiary gene pool and might be utilized for grasspea improvement by using bridge species and tissue culture techniques to break crossability barriers. The literature presents some ambiguity regarding the progenitor of *L. sativus*. However, species such as *L. cicera*, *L. marmoratus*, *L. blepharicarpus*, and *L. pseudocicera* are considered probable candidates based on their morphology and relatedness to the cultivated form [21].

**Table 1:** Classification and distribution of the genus *Lathyrus* [29].

Section	Species	Important Species	Geographical Distribution
*Orobus*	54	-	Europe, West and East Asia, Northwest Africa, North and Central America
*Lathyrostylis*	20	-	Central and Southern Europe, West Asia, Northwest Africa
*Orobon*	1	-	Anatolia, Caucasia, Crimea, Iran
*Lathyrus*	33	*L. annuus*, *L. cicera*, *L. sativus*, *L. sylvestris*, *L. tingitanus*, *L. tuberosus*, *L. gorgoni*, *L. hirsutus*, *L. latifolius*, *L. odoratus*, *L. rotundifolius*, *L. blepharicarpus*	Europe, Canaries, West and Central Asia, North Africa
*Pratensis*	6	*L. pratensis*	Europe, West and Central Asia, North Africa
*Aphaca*	2	*L. aphaca*	Europe, West and Central Asia, North Africa
*Clymenum*	3	*L. clymenum*, *L. ochrus*	Mediterranean
*Orobastrum*	1	-	Mediterranean, Crimea, Caucasia
*Viciopsis*	1	-	Southern Europe, Eastern Anatolia, North Africa
*Linearicarpus*	7	-	Europe, West and Central Asia, North and East Africa
*Nissolia*	1	-	Europe, West and Central Asia, Northwest Africa
*Neurolobus*	1	-	West Crete
*Notolathyrus*	23	-	Temperate South America, Southeast USA

## 4. Conservation of Wild Relatives of *L. sativus*

The escalating concerns of climate change, genetic erosion, and rising global demand for food production pose a significant challenge to the preservation, characterization, and utilization of *Lathyrus* plant genetic resources in various geographical regions. Global germplasm collections have huge genetic variability of both cultivated and wild species which have been assembled and maintained ex situ at various national and international institutes (Table 2). In 2016, the International Center for Agricultural Research in the Dry Areas (ICARDA) inaugurated a new crop genebank in Lebanon which holds 29% of the world’s *Lathyrus* collections from around the world. This collection stands out for its unique composition, with 45% and 54% of the accessions being wild relatives and landraces, primarily of *L. sativus*, followed by *L. cicera* and *L. ochrus*. The largest collections of *Lathyrus* are held by the Conservatoire botanique national Midi-Pyrénées, France (4477), ICARDA, Lebanon (4468), the Indian Council of Agricultural Research National Bureau of Plant Genetic Resources, India (2622), and the Bangladesh Agricultural Research Institute, Bangladesh (2422). The global conservation strategy for *Lathyrus* emphasizes the importance of enhancing documentation systems, ensuring safe multiplication and duplication, and adopting international standards to manage existing collections [44]. These measures are crucial in establishing a rational and effective conservation system for grasspea germplasm. As part of a global backup strategy, the Svalbard Global Seed Vault stores 4510 accessions from 18 depositors. These accessions come from diverse regions and belong to 45 different *Lathyrus* species (https://seedvault.nordgen.org/ accessed on 15 October 2024). In the last few decades, the creation of the *Lathyrus* Genetic Resources Network [45] and Global Crop Diversity Trust laid the groundwork for organized international conservation, collection, and other pre-breeding work on *Lathyrus*. Large collections, comprising over 800 accessions of *L. cicera* and *L. odoratus* and fewer collections of other *Lathyrus* species can be found in several countries [46,47]. The International Plant Genetic Resources Institute (IPGRI) compiled *Lathyrus* germplasm in different countries to stimulate interest in conservation and utilization and paved the pathway for research and development in this underutilized genus [45]. Genesys (online global portal) contains over 15,208 accessions of *Lathyrus* (as of 14 August 2024), which are stored in various genebanks worldwide. Most of these accessions are of *L. sativus* (8005), *L. cicera* (1242), *L. aphaca* (783), *L. ochrus* (616), *L. inconspicuus* (373), *L. hirsutus* (356), *Lathyrus* sp. (327), *L. clymenum* (280), *L. hierosolymitanus* (262), and *L. annus* (241) (accessible at www.genesys-pgr.org accessed on 24 August 2024). Many of them are duplicates and need proper phenotypic and genotypic characterization. Moreover, it is imperative to explore the genetic diversity within these collections to fully comprehend their potential use and identify key gaps. Nevertheless, the considerable sizes of many of these collections, both individually and collectively, pose challenges for characterizing, assessing, and maintaining them, impeding their effective utilization [48].

In-situ conservation of *Lathyrus* has received relatively less attention, and local populations are vulnerable to genetic deterioration or even extinction. Gap analyses have been suggested to direct upcoming collections of *Lathyrus* as well as to focus in situ preservation initiatives [47]. A gap analysis of ex situ conservation revealed that only 6 out of 37 priority species have been sufficiently sampled, underscoring the need for additional collection missions in the under-represented areas and the collection of closely related wild taxa [44]. Furthermore, six priority species lack any ex-situ collections, necessitating focused collection missions. Surveys based on eco-geographical regions indicated that the conservation efforts should prioritize *L. sativus*, *L. cicera*, *L. ochrus*, and other relevant species within their natural habitats [44]. The highest genetic diversity within the grasspea was identified in the Near East and North Africa, making these regions essential for further investigation. However, the often allogamous nature of grasspea has significant implications for collecting and multiplying genetic resources for conservation and future breeding [20].

**Table 2:** The collections of *Lathyrus* in major global genebanks (modified and updated from [49]).

Scheme	Major Genebanks	Total Accessions (with Three Major Species)
1	Conservatoire botanique national Midi-Pyrénées (CBNPMP), France ^#^	4477
2	International Center for Agricultural Research in Dry Areas, Lebanon (ICARDA) *	4468 (*L. sativus*—2577, *L. aphaca*—346, *L. cicera*—216)
3	Indian Council of Agricultural Research National Bureau of Plant Genetic Resources (ICAR-NBPGR), New Delhi, India **	2767
4	Bangladesh Agricultural Research Institute (Plant Genetic Resource Centre (BARI-PGRC)), Bangladesh ^#^	2422 (*L. sativus*)
5	Instituto Nacional de Investigación Agraria (INIA), Chile ^#^	1824
6	Australian Grains Genebank, Australia *	1477 (*L. sativus*—896, *L. cicera*—201, *L. ochrus*—122)
7	Millennium Seed Bank (MSB), Kew, England *	1103 (*L. aphaca*—196, *L. sativus*—155, *L. hierosolymitanus*—86)
8	Ustymivka Experimental Station of Plant Production, Ukraine *	1215 (*L. sativus*—782, *L. cicera*—73, *L. hirsutus*—70)
9	N.I. Vavilov All-Russian Scientific Research Institute of Plant Industry, Saint Petersburg, Russia *	1207 (*L. sativus*—824, *L. cicera*—86, *L. hirsutus* —45)
10	United States Department of Agriculture (USDA) National Plant Germplasm System *	864 (*L. sativus*—294, *Lathyrus sp*.—120, *L. odoratus*—52)

^#^ [50]. * https://www.genesys-pgr.org/a/overview/v2Vd8B228KX (accessed on 24 August 2024). ** http://genebank.nbpgr.ernet.in/SeedBank/CropSpecieswithICECWise.aspx?CropCode = 1641 (accessed on 15 October 2024).

## 5. β-ODAP: A Persistent Barrier for Grasspea Improvement

β-ODAP is a plant active ingredient, soluble in water and an analog of the proteinogenic amino acid L-glutamic acid [51]. It was first isolated from the seeds of *L. sativus*, with hemostatic and anti-inflammatory effects [52], and later was also reported in 20 species of *Lathyrus*, 17 of *Acacia*, and 13 of *Crotalaria* [53]. The grasspea toxin is present in two isomeric forms, α and β, with the β-isomer accounting for approximately 95% of the total toxic ODAP content [54,55]. While the plant’s relative tolerance against both biotic and abiotic stresses may be linked to the presence of β-ODAP, ongoing research has yet to provide conclusive evidence to support this hypothesis. β-ODAP (β-N-oxalyl-L-α,β-diaminopropionic acid) is a neurotoxic compound found in grasspea (*L. sativus*) that has been associated with lathyrism, a neurological disorder causing paralysis of the lower limbs. Research indicates that the accumulation of β-ODAP in grasspea may be associated with levels of total free nitrogenous compounds, with nitrogen and phosphate being critical factors affecting neurotoxin content under field conditions [56]. Some studies have suggested that deficiencies in cysteine and methionine may exacerbate β-ODAP’s neurotoxicity [56]. Although the full biosynthetic pathway of β-ODAP is yet to be identified, it has been proposed that β-ODAP production may be linked to sulfur metabolism. However, our understanding of sulfur metabolism and its role in ODAP biosynthesis in grasspea remains limited [57]. Additionally, an inverse relationship has been observed between certain amino acids, such as serine and cysteine, and β-ODAP accumulation, with β-cyanoalanine synthase emerging as a key enzyme in the process [58].

The presence of β-ODAP in grasspea has been one of the major limitations in its widespread use as a food source, especially during times of famine when it becomes a staple in some regions. Several grasspea varieties with reduced β-ODAP content have been developed through selection from natural germplasm and classical breeding and have been released in countries such as India, Bangladesh, Australia, and Ethiopia [3,59,60,61]. However, to eliminate the risk of neurolathyrism and fully harness the potential of grasspea, developing varieties with extremely low (below 0.1% of seed weight) or zero β-ODAP levels, irrespective of environmental factors, remains a crucial breeding goal for the ICARDA and countries like Bangladesh and Ethiopia, which heavily rely on this crop. Despite the efforts, no cultivar has yet developed with zero β-ODAP content. Efforts have been made toward understanding the biosynthesis and genetics of β-ODAP, but there are still significant gaps in our understanding of the production of β-ODAP in grasspea. The lack of a suitable animal model for neurolathyrism and the absence of a defined safe threshold for β-ODAP consumption complicate efforts to address the disease. One potential solution to address the challenge of high β-ODAP levels in grasspea is the use of CWRs to develop low-ODAP varieties. CWRs often possess genetic traits not found in the cultivated gene pool, such as natural tolerance to stresses and lower ODAP content. In the following sections, we explore the use of CWRs in the improvement of *Lathyrus* species. By leveraging the genetic diversity found in CWRs, breeding programs are making significant strides toward reducing ODAP content and enhancing the overall safety and viability of grasspea as a food crop.

## 6. Pre-Breeding for Accessing Novel Alleles from Wild Species for Crop Improvement

Pre-breeding provides interesting opportunities to harness genetic diversity by introducing desirable traits and genes from CWRs into cultivated crops by using innovative techniques. Wild species offer a wealth of valuable traits, including disease resistance and environmental adaptability, making them promising contributors to crop resilience. Despite the well-recognized potential of CWRs, their underutilization in breeding programs remains a matter of concern. This requires focused research on cross-compatibility barriers between cultivated and wild species, disparities in ploidy levels, linkage drags, and issues related to the poor viability and sterility of F_1_ hybrids, and the identification and management of desirable recombinants in subsequent generations. A prerequisite for engaging in pre-breeding initiatives is the identification of advantageous traits and/or genes within unadapted germplasm, encompassing exotic landraces or wild species. Through strategic hybridization, one strives to introgress a significant frequency of these advantageous genes and alleles into an elite background. In comparison to conventional breeding, pre-breeding populations often are assessed to pinpoint valuable introgression lines (ILs) featuring traits or genes from wild species. These ILs, chosen for their acceptable agronomic backgrounds, can then be made available for breeders to use in their ongoing breeding programs. This approach ensures a consistent infusion of diverse genetic variability into the breeding pipeline, facilitating the development of new cultivars with a broad genetic base.

## 7. Role of CWRs in Grasspea Improvement

A significant portion of a crop’s genetic diversity can come from its CWRs which have remained unaffected by domestication. CWRs can play crucial roles in the genetic enhancement of domesticated species, providing valuable traits and genetic resources that can contribute to crop improvement. Unlike the cultivated gene pool, landraces and wild relatives retain functional and adaptive genetic variation [62]. In the case of grasspea, the use of CWRs has been critical for further domestication and cultivation of *L. sativus* for sustenance, particularly to develop low-ODAP cultivars with high biological yield (Table 3). Some wild relatives, i.e., *L. cicera*, *L. amphicarpus*, and *L. ochrus*, species with almost no β-ODAP content (≤0.01%), can be utilized for the development of toxin-free grasspea varieties [21,63]. In addition to having low ODAP content, *L. cicera* is also an excellent source for earliness, biomass, and cold tolerance. Screening of *L. aphaca*, *L. cicera*, *L. blepharicarpus*, *L. cassius*, *L. pseudocicera*, and *L. ochrus* has also identified sources of resistance to broomrape [64,65,66,67], indicating utilization of CWRs in introgression breeding for grasspea improvement. In addition, Vaz Patto and Rubiales [68] also identified *L. cicera* accessions with resistance to powdery mildew and rust. Other pertinent reports include resistance to the northern root-knot nematode (*Meloidogyne hapla*), and *Pseudomonas syringae* pv. *Syringae* in *L. latifolius*, *L. sylvestris*, and *L. hirsutus* [69].

**Table 3:** Potential sources of crop wild relatives for grasspea improvement.

Species	Trait	Reference
*L. aphaca*	Broomrape (*Orobanche crenata* and *O. foetida*)	[67]
*L. cicera*	Zero or low ODAP (0.01% or less)	[3,63]
	Bruchid	[70]
	Earliness and cold tolerance	[71]
	Low ODAP, earliness, and cold tolerance	[3,72,73]
	*Pseudomonas syringae*	[69]
	Broomrape (*Orobanche crenata* Frosk)	[65,66]
*L. clymenum*	Crenata broomrape (*Orobanche crenata*)	[64]
*L. cicera*, *L. odoratus*	Pod borer	[45]
*L. hirsutus*, *L. latifolius*, *L. sylvestris*	Root-knot nematode (*Meloidogyne artiella*)	[74]
*L. cicera*, *L. ochrus*, *L. amphicarpus*	Zero or low ODAP	[21,63]
*L. ochrus*	Broomrape (*Orobanche crenata*)	[64,75]
	Broomrape *(Orobanche crenata* and *O. foetida)*	[67]
*L. ochrus*, *L. clymenum*	Ascochyta blight (*Mycosphaerella pinodes*)	[76,77]
*L. sativus*	Powdery mildew (*Erysiphe pisi* and *E. trifolii*)	[69]
	*Cercospora pisi-sativae*	[78]
*L. sativus*, *L. cicera*	Powdery mildew (*Erysiphe pisi*)	[7,79,80]
	Rust *(Uromyces pisi*)	[81,82]
*L. tingitanus*	Drought tolerance Heat tolerance High protein content Low ODAP content	[3,83]
*L. czeczottianus*	High phenolic content	[84]
*L. nissolia*	Enzymatic inhibitory effects against cholinesterase, amylase, and glucosidase	[84]
*L. sphaericus*	High phenolic content	[85]
	High protein content	[86]
*L. annuus*	High antioxidant activity	[85]
	High protein content	[86]

## 8. Reproductive Barriers Hindering Utilization of CWRs in Grasspea Improvement

Wide hybridization can transfer beneficial traits from related crop species or their wild relatives, but major impediments often curtail its effectiveness. Despite the successful production of viable seeds, attempts to transfer alien genes among *L. sativus*, *L. cicera*, and *L. amphicarpus* are rare [87,88,89]. Using easily crossable species like *L. cicera* and *L. amphicarpus*, alien gene transfer into *L. sativus* is possible, based on information regarding crossability, alteration of chromosome behavior in hybrids, and the successful production of viable seeds. However, there are pre- and post-fertilization barriers that restrict interspecific and intergeneric hybridization. Pre-fertilization barriers can involve a lack of pollen germination, restriction in the growth of pollen tubes, or their failure to penetrate the embryo [90]. In contrast, post-fertilization barriers result in the degradation or death of hybrid embryos or abnormal seed development [91]. Even if pollen germination and fertilization are successful, post-zygotic barriers can arise, leading to disturbances in normal seed development through embryo abortion and hindering endosperm development [92].

Pre-fertilization barriers were observed in *L. odoratus × L. chloranthus* crosses and reciprocal *L. odoratus × L. chrysanthus* crosses involving long-styled *L. odoratus* cultivars which prevented the growth of foreign pollen tubes to the base of styles. Herrick et al. [93] attempted interspecific hybridization between *L. odoratus* L. and *L. chloranthus* Boiss. to transfer the yellow flower color to *L. odoratus* L., but the reciprocal crosses yielded no viable seeds, indicating the presence of post-fertilization barriers that inhibited embryo maturation.

Cross incompatibilities in *Lathyrus* can be due to genetic incompatibility, differences in nuclear DNA content [94], and the presence of the non-protein amino acid lathyrine [95], which affects pollen germination and pollen tube growth when crossing between *Lathyrus* species. Biotechnological tools, including tissue culture techniques, can be deployed to overcome significant reproductive barriers among different species [96]. Use of embryo rescue techniques is a promising avenue for achieving interspecific crosses between certain species that would otherwise fail due to embryo abortion after fertilization. Mehta and Santha [97] successfully developed plant regeneration techniques, demonstrating their efficiency for reliable grasspea plant regeneration from stem, leaf, and root explants. The regenerated plants exhibited a significant degree of somaclonal variation in their plant habit. Attempts to rescue aborted interspecific hybrid embryos through in vitro culture revealed varying responses, with successful callus formation observed in most combinations, and with mature plants regenerated from immature embryos of the cross between *L. sativus* (male parent) and *L. cicera* (female parent) but not the reciprocal [90]. Despite efforts to expand the range of successful interspecific crosses via embryo rescue and protoplast fusion protocols, these methods have not yet been widely adopted for grasspea improvement [98]. The challenges lie in improving the efficiency of regenerating hybrid plants, hindering the practical integration of this approach in breeding initiatives.

## 9. Interspecific Hybridization

Interspecific hybridization is employed to introduce novel alleles into cultivated species, broadening their genetic diversity and potentially enhancing desirable traits. The success of interspecific crosses depends on various factors, such as the physical proximity of the parent plants, time of flowering, specific parental genotypes, pollen transfer methods, direction of the cross (female parent), environmental conditions, and presence of male sterility in one parent [99,100].

Several successful instances of interspecific crosses within *Lathyrus* have been documented which can serve as a viable base for grasspea pre-breeding. Many researchers have attempted to achieve interspecific hybridization within the genus *Lathyrus* following Barker’s [101] successful crossing of *L. hirsutus* and *L. odoratus*. In 1956, Lwin [102] accomplished the hybridization of *L. cicera* with *L. sativus*; nevertheless, subsequent attempts to replicate this cross proved unsuccessful [87,103]. According to Khawaja [87], *L. sativus* readily forms hybrids with *L. amphicarpus* when the latter is employed as the female parent. Other successful interspecific hybrids have been obtained from the crosses *L. rotundifolius* × *L. tuberosus* and *L. sylvestris* × *L. latifolius* [103,104,105], *L. hirsutus* × *L. odoratus* [87,106,107], *L. marmoratus* Boiss. × *L. blepharicarpus*, *L. gorgoni* × *L. pseudocicera*, *L. cicera* × *L. marmoratus*, *L. cicera* × *L. blepharicarpus*, *L. cicera* × *L. gorgoni*, *L. cicera* × *L. pseudocicera* [41,105,106], *L. cicera* × *L. clymenus* and *L. cicera* × *L. ochrus* [108,109], and *L. annuus* × *L. hierosolymilanus* Boiss. and *L. odoratus* × *L. belinenesis* [107,108]. Yunus and Jackson [40] reported interspecific hybridization involving *L. sativus* and 15 wild *Lathyrus* species and obtained viable F_1_ hybrids with reduced fertility from the cross between *L. sativus* (as male parent) and *L. amphicarpus* and *L. cicera*. Yunus [88] attempted interspecific hybridization using 11 wild species and *L. sativus*, but only an *L. cicera* × *L. amphicarpus* cross yielded viable seeds. Abdallah et al. [67] investigated the crossability of *L. sativus* with six wild species, resulting in viable seeds with *L. cicera* and, to a lesser extent, with *L. ochrus*, *L. inconspicuous*, *L. marmoratus*, and *L. heirosolymitanus*. Many *Lathyrus* species, such as *L. odoratus*, are cultivated as garden plants, and there is considerable interest in exploring the secondary gene pool in *L. odoratus* for the development of new pigmentations and scents. *L. odoratus* has been successfully crossed with *L. hirsutus*, *L. chlorantus* [87], and *L. belinensis* [109,110].

The *Lathyrus* gene pool presents significant potential as a valuable source of economically important traits, including low or zero ODAP content, enhanced resistance to biotic and abiotic stresses, and traits suitable for ornamental purposes. The genetic diversity observed in wild *Lathyrus* species offers substantial potential for improving grasspea through pre-breeding efforts. At the ICARDA, the grasspea program has benefited from the use of CWRs by generating interspecific hybrids between *L. sativus* and other *Lathyrus* species, demonstrating successful interspecific crosses with *L. cicera*, followed by crosses with *L. ochrus*, and, to a lesser extent, crosses with *L. inconspicuous*, *L. marmoratus*, and *L. heirosolymitanus*, through embryo rescue using B5 media (unpublished CWR project report). In addition, more than 100 fixed pre-bred lines emanating from *L. sativus* and *L. cicera* crosses were successfully made and evaluated at the ICARDA. Out of these, twenty-one CWR lines were selected based on their low ODAP content and high grain yield under the BOLD (Biodiversity for Opportunities, Livelihoods, and Development) project, funded by the Crop Trust, to assess their performance for yield, stability, and ODAP content during the 2023–2024 cropping season across four countries: Bangladesh, India, Nepal, and Morocco (in progress).

**Figure 1:**
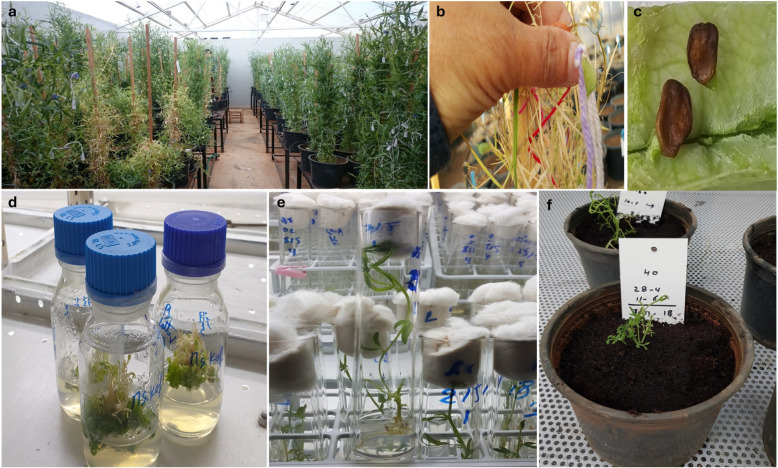
(**a**) Interspecific hybridization between *L. sativus* and *L. cicera;* (**b**,**c**) formation of pods with shriveled seed, indicating unsuccessful development; (**d**,**e**) in vitro hybrid plants successfully regenerated on MS media for embryo rescue; and (**f**) hybrid plants acclimatized in ex vitro (pots filled with soil) conditions.

To effectively harness the genetic diversity of the *Lathyrus* gene pool, it is crucial to address the reproductive barriers that exist between species. Modern biotechnological techniques, such as tissue culture, somaclonal variation [111], and protoplast fusion, may play a vital role in facilitating these interspecific crosses [96,112]. Consequently, the development of reliable and reproducible protocols is essential for the successful application of genetic transformation techniques [113,114]. Interspecific hybrids between *L. sativus* and *L. cicera* were produced at the ICARDA (CWR project report 2020) through an embryo rescue that involved the isolation and culture of embryos at a stage where they might fail to develop under normal conditions (Figure 1). The implementation of this protocol effectively overcame fertilization barriers, resulting in successful interspecific crosses. Techniques including use of 2,4-D and GA3 were employed to address fertilization incompatibility, although the initial pod yield was low at 5.9%. However, modifying the protocol by using honey to weld both wings raised humidity levels within the flowers, contributing to an increased pod yield of 15.7%. After pollinating 576 florets, the embryo rescue protocol was applied to rescue young embryos and successfully saved 33 embryos. These embryos were then transferred to a special nutrient-rich medium containing B5 and Gamborg nutrients. From these rescued embryos, we successfully grew 19 hybrid plant, demonstrating the effectiveness of the embryo rescue protocol in overcoming post-zygotic barriers in *Lathyrus* species.

The challenges of interspecific hybrids become more significant as the phylogenetic distance between the parent species increases [115]. In interspecific crosses, a phenomenon called the “one-way reproductive barrier” often occurs, where successful hybridization is observed when one species is used as the maternal parent but not in the reciprocal direction [92]. *Lathyrus cicera*, *L. nissola*, *L. stenophyllus*, and *L. tingitanus*, when crossed with *L. sativus*, developed pods when *L. sativus* is used as female parent. However, the viable seeds did not develop as the pods dried prematurely [89]. Addis and Narayan [89] attempted 12 reciprocal crosses and only 1 cross, *L. sativus* (male parent) and *L. amphicarpus* (female parent), produced viable seeds. These findings align with those of other researchers [116], confirming the difficulties in making interspecific hybrids in *Lathyrus* [116]. Nandini et al. [117] produced interspecific hybrids involving *L. latifolius*, *L. sylvestris*, and *L. heterophyllus* (all perennial species). The observation of pachytene pairing within spread synaptonemal complexes indicated that the reduced fertility in these hybrids is unlikely to be due to structural difference in chromosomes between the parental species [117].

## 10. Novel Tools and Technologies in Unraveling the Potential of Crop Wild Relatives

### 10.1. Molecular Phylogenetic Relationships Among Lathyrus

Cytologically, *Lathyrus* species differ widely in chromosome number, but most have basic (x) and somatic (2n) chromosome numbers of 7 and 14, respectively [118]. The ribulose-1,5-bisphosphate carboxylase/oxygenase large subunit (*rbcL*) gene, the non-coding *trnH-psbA* intergenic spacer of chloroplast DNA, and the internal transcribed spacer (ITS) gene region of nuclear ribosomal DNA are often employed in molecular phylogeny studies to address the questions in phylogenetic reconstruction and classification [119,120,121]. Various studies have used these methods to resolve phylogenetic issues within the genus *Lathyrus* [38,122,123,124]. Asmussen and Liston [38] studied the cytoplasmic diversity of 42 species of *Lathyrus*, whereas Kenicer et al. [39] used cpDNA sequences for a biogeographical study.

Utilizing storage protein gene sequences, Sáenz de Miera et al. [125] demonstrated that species from the section *Lathyrus* (*L. sativus*, *L. annuus*, *L. cicera*, and *L. tingitanus*) form a monophyletic group, whereas species from the section *Clymenum* (*L. clymenum* and *L. ochrus*) along with *L. latifolius* from the section *Lathyrus* form another distinct group. Kenicer et al. [39] examined the systematics and biogeography of 53 *Lathyrus* species via their nuclear ribosomal and chloroplast DNA. Their results elucidated clades between *Lathyrus* and *Lathyrostylis* sections and generally confirmed the current categorization based on morphological traits. However, they also raised doubts regarding the monophyly of the section *Orobus sensu* Kupicha [37]. Ceccarelli et al. [126] used satellite DNA to confirm the close phylogenetic relationship between *L. sylvestris* and *L. latifolius* first reported by Asmussen and Liston [38] based on chloroplast DNA. Oskoueiyan et al. [120] used matK as a selected barcode to construct the phylogeny of 60 species of *Lathyrus*. Their findings also concurred with Asmussen and Liston [38], confirming that the sections *Orobon* and *Lathyrus* should be combined. They also reported that *L. clymenum* and *L. ochrus* (section *Clymenum*) are closely related to taxa.

The existing taxonomic categorization of *Lathyrus* has been supported by ITS, nuclear ribosomal, and chloroplast (cp) sequence-specific DNA markers [39]. Among the most frequently utilized nuclear markers in plants are those from the ITS region of nrDNA, particularly ITS2. Marghali et al. [97] used ITS2 sequence data to study the molecular evolution of *Lathyrus* spp., revealing that *L. aphaca* had the largest genetic distances from all other species, with the greatest genetic divergence observed between *L. angulatus* and *L. aphaca*. Similarly, Asmussen and Liston [38] noted that the *Linearicarpus* section, as represented by *L. sphaericus* and *L. angulatus*, is clearly distinct from the section *Aphaca*. *Lathyrus* species pairs with high similarity in ITS2 sequences include pairs such as *L. setifolius* and *L. tuberosus*, *L. gorgoni* and *L. tuberosus*, *L. setifolius* and *L. gorgoni*, and *L. latifolius* and *L. tuberosus*. Levels of sequence divergence among *Lathyrus* species seem to be linked to their high rate of evolutionary change. Marghali et al. [123] used Maximum Parsimony (MP) and Neighbor-Joining (NJ) methods to explore genetic relationships among *Lathyrus* species by building phylogenetic trees. The MP analysis of ITS2-nrDNA revealed a range of genetic variation among *Lathyrus* species and supported lineages within the *Vicieae* clade. The NJ method grouped the species into two clusters: Cluster 1, which included *L. sylvestris* and *L. aphaca* from two different sections (*Lathyrus* and *Aphaca*), and Cluster 2, which comprised all other species. However, Marghali et al. [123] found that the ITS2 phylogeny does not fully align with Kupicha’s classification and does not support the close relationship between the *Pratensis* and *Aphaca* sections proposed by Kupicha [37]. Additionally, their ITS2 tree indicated a close relationship between *L. sativus* and *L. cicera*, corroborating previous analyses based on morphology and plastid DNA or nuclear markers, and suggests successful interspecific hybridization between these two species [102].

### 10.2. Molecular Markers and Genetic Diversity

The availability of molecular markers in grasspea is currently quite limited, highlighting the need to address this gap to successfully develop molecular breeding strategies. An effort has been made to use EST-SSR markers (expressed sequence tags–simple sequence repeats) from closely associated legume species to overcome the limitation. The development of genic SSRs (EST-SSRs) in *L. sativus* and *L. cicera* has been facilitated by the availability of cross-species amplified markers and computational searches of the nucleotide sequence database of ESTs available at NCBI GenBank [127,128]. Additionally, by analyzing the RIL population of *L. cicera* and sequencing the monomorphic simple sequence repeat (SSR) segments, cleaved amplified polymorphic sequences (CAPS) and derived CAPS (dCAPS) were also developed [129]. Recently, a panel of SNP-derived kompetitive allele-specific PCR (KASP) markers and a set of polymorphic EST-SSRs were developed by RNA-sequencing-based transcriptome analysis using the Illumina NextSeqTM500 platform [130]. The development of rapid genome-wide SSRs and single-nucleotide polymorphism (SNP) detection, which should facilitate positional cloning and QTL mapping, has been made possible by the advancement of next-generation DNA sequencing technology in the absence of a reference genome for grasspea (Table 4) [131]. There are only a few reports on the development of genomic SSR markers for *Lathyrus* [132,133,134,135]. Yang et al. [134] developed 50,144 unique SSRs, of which 288 were randomly selected to validate 24 accessions of *Lathyrus* (including 23 *L. sativus* and 1 *L. cicera*). The development of this large number of SSR markers is a significant advancement for the genomics-enabled improvement of grasspea.

The genetic diversity of the genus *Lathyrus* has been assessed with morphological markers [136,137], isozyme polymorphisms [138], and DNA-based markers such as random amplified polymorphic DNA (RAPD), restriction fragment length polymorphisms (RFLPs), amplified fragment length polymorphism (AFLP), inter simple sequence repeat (ISSR), and microsatellites or SSRs [139] (Table 5). ISSR analysis revealed that *L. sativus* is more closely related to *L. cicera* than it is to *L. ochrus* [140]. Such findings support the classification of *L. ochrus* into the section *Clymenum*, as proposed by Kupicha [37]. Additionally, results from RFLP and RAPD analyses indicate a close genetic relationship between *L. sativus* and *L. cicera* and *L. sylvestris* and *L. latifolius* [141]. Eleven ISSR markers were employed to assess the phylogenetic relationships and genetic diversity of four sections of the genus, *Lathyrus*, *Clymenum*, *Nissolia*, and *Aphaca*, revealing close genetic similarity between *L. sativus* and *L. cicera* [142]. In contrast, *L. ochrus* showed greater similarity to *L. aphaca* and *L. nissolia*. AFLP has also been used to assess the genetic diversity of *L. sativus* and its relatives [132,142].

Subsequently, 30 SSRs were used to analyze 266 *Lathyrus* accessions and 17 related species from Africa, Europe, Asia, and the ICARDA [135]. Clustering analysis based on Nei’s genetic distance divided species into two clusters: cluster I comprised all annual species (*L. sativus*, *L*. *cicera*, *L*. *tingitanus*, *L*. *aphaca*, and *L. hirsutus*), and cluster II comprised annual (*L*. *clymenum* and *L*. *ochrus*) and perennial species (*L*. *pratensis*, *L*. *sylvestris*, and *L*. *latifolius*). Population structure analysis of the studied accessions indicated potential gene flow between European and African accessions, which was further supported by UPGMA-based cluster analysis and principal component analysis (PCA).

The genetic diversity of three *Lathyrus* species (*L. sativus*, *L. cicera*, and *L. ochrus*) was also evaluated by using ten SSR markers [143]. Population structure analysis revealed that *Lathyrus* accessions were grouped into three distinct clusters, independent of geographic origin, with significant genetic differentiation observed between *L. ochrus* and the two species. These findings have been supported by other DNA-marker-based studies that employed RFLP [141], ISSR [140], and SSR markers [135]. Rahman et al. [144] studied the genetic diversity of *L. sativus* from different geographic regions using 56 SSR markers. Cluster analysis divided the germplasm into two main groups and one subgroup, while the model-based population structure analysis identified three populations.

EST-SSRs are highly conserved and can often be transferred among species [145]. While only 178 ESTs are available for *L. sativus* and 126 for *L. cicera* at NCBI, the number is much higher for *L. odoratus* (8702 ESTs) [11,139]. An SSR genomic enriched library was developed for *L. sativus* and tested for transferability among *Lathyrus* species. Seven SSR markers showed high transferability among three related species of *Lathyrus*, *L. cicera*, *L. ochrus*, and *L. tingitanus*, as well as to *Pisum sativum* [146]. Over the last two decades, EST-SSRs have been used repeatedly for diversity analysis of *Lathyrus* genotypes [135,147,148,149]. Numerous molecular markers from *Medicago truncatula*, garden pea, lentil, lupin, and faba bean have also been shown to be transferable to *L. cicera* and *L. sativus* for future application in mapping and diversity studies [150,151]. These results indicate that the novel SSR markers are informative and will be useful and convenient for genetic analysis in grasspea and related species. CAPS and derived CAPS (dCAPS) have also been developed for use in *Lathyrus* [128,151]. In silico mining of nucleotide sequences identified 203 SSRs, of which 150 were used for marker–trait association in 50 geographically diverse accessions of *Lathyrus* spp. from India, Syria, Ethiopia, France, Italy, Bangladesh, and Korea [152]. By using a mixed linear model, six significant marker–trait associations were identified for phenological and yield-related traits [152]. New genomic resources have enabled the use of *Lathyrus* as a valuable source of traits for related species and vice versa.

**Table 4:** Application of NGS techniques in grasspea.

Genotype Used	Trait	Sequencing Platform	No. of Primers Detected	No. of Genes Annotated	References
Eight grasspea accessions consisted of two Chinese, two Asian, one African, and three European accessions	-	Roche 454 GS FLX Titanium platform	651,827	-	[134]
BGE015746, BGE024709	Rust resistance	Illumina (San Diego, CA, USA) Hiseq2000	2634 SNPs 200 EST-SSR	50,937 (60.4% into functional categories)	[128]
BGE015746	Aschochyta blight resistance	Illumina Genome Analyser IIx	-	13,773	[153]
Rewa-2	-	Illumina HiSeq 2500	1139 SSRs	-	[154]
RQ23 and RQ36	-	Illumina NextSeq™ 500	3204 EST-SSR, 146,406 SNP	-	[130]
LZ	β-ODAP	Illumina HiSeq 3000	-	27,032	[131]
LP-24	Drought	Illumina HiSeq 2500	8079 SSRs	31,368	[155]

**Table 5:** Diversity analysis in *Lathyrus* spp. by molecular markers.

Species	Marker Type	Reference
*L. sativus*	SSR	[144,156,157,158,159]
	EST-SSR	[147,149]
	RAPD	[160]
	AFLP	[132]
	CAPS	[129]
	ISSR	[142]
*L. sativus*, *L. cicera*	SSR	[134]
*L. sativus*, *L cicera*, *L. ochrus*	SSR	[143]
	ISSR	[140]
*L. sativus*, *L. cicera*, *L. latifolius*, *L. ochrus*	RAPD	[141]
*L. sativus*, *L. cicera*, *L. aphaca*, *L. clymenum*, *L. hirsutus*, *L. ochrus*, *L. tingitanus*, *L. latifolius*, *L. pratensis*, *L. sylvestris*	SSR	[135]
	Cross transferable	
*L. sativus*	*L. sativus*- and *Lotus japonicus*-derived EST-SSR	[132]
	*M. truncatula*- and *L. sativus*-derived EST-SSR	[133]
*L. sativus*, *L. cicera*	*P. sativum*- and *M. truncatula*-derived ITAP and *P. sativum*-derived gSSR and EST-SSR	[151]

### 10.3. Genetic Linkage Maps

Molecular mapping is crucial for identifying and locating specific genes associated with desirable traits in crops. It enhances the precision and efficiency of breeding programs by enabling marker-assisted selection (MAS), accelerating the development of improved crop varieties. Over time, various genetic maps have been developed for grasspea, based on various marker types. The first linkage map was developed by using 11 RAPD markers, 1 isozyme marker, and flower color [140]. Skiba et al. [77] developed a genetic map using 47 RAPD markers, 7 cross transferable markers from pea, and 13 CAPS markers for QTL analysis of Ascochyta blight resistance in a backcross population. While QTLs linked to resistance were identified, no candidate genes were found, limiting their use in precision breeding [7]. However, neither of these linkage maps was densely populated with markers, resulting in gaps and short linkage groups (LGs). Consequently, they could not be aligned or compared effectively with linkage maps for other legumes. Fortunately, high-resolution genetic maps, crucial for modern selective breeding, have recently been developed for *L. sativus* and *L. cicera* [161,162]. Santos et al. [161] mapped 1468 markers (silicoDArT, SNPs, ESSR, and ITAP) across 9 LGs with a total length of 712.35 cM and an average interval distance of 0.65 cM. They identified three QTLs (*EpDSI*, *EpDSII*, and *EpDSIV*) for *Erysiphe pisi* resistance, exhibiting phenotypic variation from 8.2 to 13%, and one QTL (*EtDSVIII*) for *E. trifolii* reaction, explaining 16% of phenotypic variation. More recently, 2149 DArT-Seq markers were assigned to 10 LGs (7 major and 3 minor), with a total genetic distance of 674.4 cM and an average interval distance of 0.41 cM [162]. An SNP associated with *LsMLO1*, which regulates powdery mildew susceptibility, was identified on LG 1 at position 18.246 cM [162]. Association mapping studies in *L. sativus* collections have highlighted the oligogenic nature of resistance to fusarium wilt and rust [163,164]. Advancing grasspea research will require the rapid development of an even more detailed genetic map to identify valuable genes and QTLs for MAS. Comparative mapping with related species could further enhance this effort by aligning their genetic data. Creating comprehensive linkage maps, combined with gene cloning techniques and MAS, can expedite the incorporation of novel genes associated with key traits such as disease resistance and reduced ODAP content.

### 10.4. Genome Sequencing

Leveraging the extensive array of germplasm and genetic resources within *Lathyrus* critically depends on the availability of top-tier genome sequence information, which holds great promise for identifying genes in the β-ODAP biosynthetic pathway and selecting desirable traits for agronomic improvement. One notable achievement in this regard is the genome sequencing of the European grasspea cv. “LS007”. Emmrich et al. [165] produced a draft genome sequence for this cultivar with a draft genome assembly of approximately 6.3 Gbp, with an N50 of about 59.7 kbp [165], and estimated the grasspea genome to be within the range of 5.456–8.471 Gbp. Such genomic data are essential for understanding the intricate biosynthetic pathway responsible for producing β-L-ODAP. Edwards et al. [166] further presented a comprehensive, annotated, long-read-based assembly of a 6.5 Gbp *L. sativus* genome which decoded the biosynthetic pathway for formation of β-L-ODAP. Additionally, Rajarammohan et al. [167] reported a high-quality reference assembly of *L. sativus* cv. ‘Pusa-24’. Their assembled genome, approximately 3.80 Gb in length, boasted a scaffold N50 of 421.39 Mb, indicating excellent contiguity. Furthermore, the assessment of highly conserved Viridiplantae genes revealed an impressive 98.3% presence in the assembly. The identification of 3.17 Gb (83.31%) of repetitive sequences and 50,106 protein-coding genes in the *Lathyrus* assembly further enriches our understanding of its genomic composition. A draft genome assembly also has been developed for *L. tuberosus*, based on Pacific Biosciences sequence reads [168]. Overall, these groundbreaking achievements in genome sequencing offer great potential for harnessing the diverse genetic resources within *Lathyrus* spp., developing marker sets for use in breeding programs, and assessing the genomic effects of domestication, and identifying key loci that differentiate wild and cultivated taxa.

### 10.5. Omics Approaches

Transcriptome studies have primarily aimed to generate a vast array of genome-wide SSRs and SNPs for applications in molecular mapping and map-based cloning research. Recent research in grasspea has focused on transcriptomics [128,130,134,154,169]. A few studies have focused on identifying genes and mechanisms regulating biotic and abiotic stresses, as well as the metabolic pathway that controls β-ODAP in grasspea [131]. Also, transcriptome studies have been carried out to find the genes and regulatory pathways that control the β-ODAP flux in grasspea at various developmental stages [155]. To determine differential proteomes in response to salt and low-temperature stress, a comparative proteomics analysis was carried out in grasspea that identified 67 differentially regulated proteins [58,170].

### 10.6. Genome Editing

Genome editing has been used in model plants, crops, and fruits [171,172]. It enables precise, targeted modifications in a plant’s DNA. The toolbox for CRISPR/Cas9 can be used to modify the genome in ways that can broaden the gene pool and allow the rapid generation of new varieties. The concentration of sulfur-containing amino acids like methionine and cysteine has been successfully increased using CRISPR/Cas9 [173]. The amount of β-ODAP in grasspea can be reduced through a change in the concentration of methionine [56]. The successful use of this cutting-edge technique has been hampered by the fact that grasspea is known to be resistant to the absorption and integration of foreign DNA and to regeneration. However, researchers are working to broaden the CRISPR/Cas9 system in the grasspea improvement program for the engineering of signaling pathways or regulatory mechanisms engaged in environmental stresses and ODAP biosynthesis [174]. Bekele-Alemu et al. [175] proposed four strategies to reduce or eliminate the biosynthesis of β-ODAP in grasspea. The first approach focuses on targeting the β-ODAP synthase (*BOS*) gene alone, while the second involves targeting the cyanoalanine synthase (*CAS*) gene independently. The third strategy suggests simultaneously targeting both *BOS* and *CAS* genes. Lastly, the fourth approach involves modifying specific amino acids in either the *BOS* or *CAS* genes, or both, to partially reduce β-ODAP production. This final strategy is recommended if a complete or partial knockout of β-ODAP biosynthesis results in undesirable effects, such as increased sensitivity to abiotic stresses like drought. In the first strategy, which focuses on targeting the *BOS* gene, it is also essential to account for the potential accumulation of the precursor L-DAPA and monitor levels of any toxic intermediates. The in vitro toxicological effects of isoxazoline amino acids found in *L. sativus* were previously studied by [176,177]. Additionally, Verma et al. [178] demonstrated the gene editing of the *LsOCS* gene involved in oxalylation in ODAP biosynthesis and established a hairy root transformation system for *Lathyrus*. Through their groundbreaking and functional analysis of this gene, there is hope that variants from CWRs can be transformed into ideal future crops by using precise gene-editing technologies in combination with developing sequence data from wild genomes.

## 11. Conclusions

The improvement of *L. sativus* through the incorporation of CWRs offers a promising pathway to address critical challenges such as reducing ODAP toxicity, enhancing resistance to biotic and abiotic stresses, and improving agronomic traits. The origin, domestication, and geographic distribution of grasspea reveal a rich genetic diversity within this species, and particularly within its wild relatives. Taxonomically, the gene pool of *Lathyrus* species provides a wealth of untapped alleles that could significantly contribute to crop improvement. To fully harness these genetic resources, it is essential to focus on addressing critical gaps in genebank collections and improving seed conservation strategies, particularly for CWRs. These efforts will not only ensure the preservation and enrichment of valuable genetic material but also guide future germplasm collection and expedition strategies. Additionally, they will support long-term initiatives like the CWR Project, which aim to prevent genetic erosion and maintain biodiversity for future breeding efforts. Conservation efforts focused on CWRs are crucial, as they provide a reservoir of novel alleles that can be accessed through pre-breeding efforts. These alleles are key to enhancing traits such as low ODAP content, stress resistance, and yield stability. However, the utilization of CWRs is often hampered by reproductive barriers, which limit the success of interspecific hybridization. Despite these challenges, modern tools such as embryo rescue, somaclonal variation, and protoplast fusion have shown promise in overcoming these barriers and facilitating gene flow between species. Moreover, when combined with advanced genomics tools and modern breeding technologies, these approaches offer a comprehensive strategy for grasspea improvement, enhancing the efficiency of incorporating beneficial traits from crop wild relatives. Genomic approaches, such as the identification of key genes and QTLs through linkage mapping and MAS, have the potential to accelerate the incorporation of novel alleles from CWRs into cultivated varieties. The rapid advancements in omics can offer detailed insights into genetic, biochemical, and physiological diversity across growth stages. Furthermore, cutting-edge genome-editing technologies, such as CRISPR/Cas9, offer transformative possibilities by targeting genes responsible for undesirable traits like ODAP synthesis, paving the way for safer and more productive grasspea varieties. By leveraging these innovations alongside traditional breeding methods, the potential of CWRs can be fully realized, contributing to the development of grasspea varieties with enhanced resistance, reduced ODAP levels, and improved agronomic traits.

## Data Availability

The data presented in this study are available in the manuscript.
